# Cutaneous parasitism in patients with American visceral leishmaniasis in an endemic area

**DOI:** 10.1590/0037-8682-0446-2019

**Published:** 2020-02-21

**Authors:** Carla Riama Lopes de Pádua Moura, Carlos Henrique Nery Costa, Rafael de Deus Moura, Aline Reis Ferro Braga, Vladimir Costa Silva, Dorcas Lamounier Costa

**Affiliations:** 1 Universidade Federal do Piauí, Departamento de Medicina Especializada, Teresina, PI, Brasil.; 2 Universidade Federal do Piauí, Instituto de Doenças Tropicais Nathan Portella, Teresina, PI, Brasil.; 3 Universidade Federal do Piauí, Hospital Universitário, Laboratório de Anatomia Patológica, Teresina, PI, Brasil.; 4 Laboratório de Pesquisa em Leishmanioses, Teresina, PI, Brasil.

**Keywords:** Visceral leishmaniasis, Leishmania infantum, Kala-azar, Skin, HIV

## Abstract

**INTRODUCTION:**

Visceral leishmaniasis (VL) represents a public health concern in several areas of the world. In the American continent, VL transmission is typically zoonotic, but humans with active VL caused by *Leishmania infantum* are able to infect sandflies. Thus, individuals with cutaneous parasitic infections may act as reservoirs and allow interhuman transmission. Additionally, the skin may be responsible for reactivation of the disease after therapy. This study’s objective was to evaluate cutaneous parasitism in humans with VL in an American endemic area.

**METHODS:**

A cross-sectional hospital-based study was conducted in northeast Brazil from October 2016 to April 2017. Biopsies of healthy skin for histopathology and immunohistochemistry were performed prior to treatment in all study patients.

**RESULTS:**

Twenty-two patients between the ages of five months to 78 years were included in the study. Seven patients (31.8%) tested positive for HIV. Only one patient had cutaneous parasitism, as confirmed by immunohistochemistry prior to treatment. Parasitism was not detected after treatment.

**CONCLUSIONS:**

Cutaneous parasitism in the healthy skin of humans with visceral leishmaniasis, although unusual, may be a source of infection for phlebotomine sandflies.

## INTRODUCTION

Visceral Leishmaniasis (VL) is an endemic disease undergoing geographical expansion and represents a public health concern in many regions of the world^1^. In Asia and East Africa, VL is caused by *Leishmania donovani*, while in the American continent and Southwestern Europe the main infectious agent is *L. infantum.* The transmission can be anthroponotic, zoonotic, or both, depending on the endemic area^2^. The main vector for *L. infantum* in the American continent is *Lutzomyia longipalpis* ^3^, a sandfly species. In urban areas, the domestic dog is considered to be the main source of infection for the vector^1^, and as far as we know, little has been published on interhuman transmission in Brazil^4^ ^,^ ^5^ ^,^ ^6^ ^,^ ^7^.

Programs focusing on disease control usually include vector control, culling sick dogs and early treatment of affected humans^1^. However, the elimination of infected dogs has been ineffective^3^ ^,^ ^8^ suggesting that other competent reservoirs of *L.infantum* ^5^ may exist. 

Silva *et al.* ^9^ observed an extreme difference in parasitemia when it was estimated by qPCR or measured by direct microscopy; parasitemia was not mathematically compatible with the proportions of infected insects in published xenodiagnostic studies. This group proposed that skin, rather than blood, could be the main source of infection for the vectors.

A study with minimally invasive micro-biopsies, which were aimed at identifying asymptomatic and potentially infectious *L. donovani* carriers, found that the skin, more often than the blood, was the source of parasites. The reason for this was that although the volume of the micro-biopsies was ten times smaller than finger prick blood samples, the rates of detection of *Leishmania* DNA in micro-biopsies was significantly higher^10^. In addition, cutaneous parasitism in apparently healthy skin samples has been observed in humans with active VL^11^ ^,^ ^12^ ^,^ ^13^ ^,^ ^14^.

Considering the possible epidemiological implications of cutaneous parasitism in humans, which may facilitate its transmission and be responsible for post-therapy reactivation of the disease, we conducted a study in search of parasites living on the healthy skin of people with American visceral leishmaniasis in an area endemic for *L. infantum*.

## METHODS

### Type of study and place of conduction

A cross-sectional study was performed with the selection of participants with a diagnosis of Visceral Leishmaniasis. Participants were of both genders and all ages and were recruited in Teresina, Northeast Brazil.

A patient was considered to have VL if they presented with the typical symptoms of fever, wasting, paleness and hepatosplenomegaly with confirmation by at least one of the following tests: direct visualization of amastigotes in tissues, visualization of promastigote forms in culture, immunochromatographic test with rK39 antigen (IT- LEISH^®^) or polymerase chain reaction (PCR). Pregnant or breastfeeding women, patients with leishmaniasis-like skin wounds, patients with an absence of healthy skin regions on the forearms due to other skin conditions such as ichthyoses and patients who had received any anti-*Leishmania* drug in the last 6 months were excluded from the study.

Skin biopsy was performed on the back of the forearm using a 3 mm punch, and the cutaneous fragment was fixed in 10% neutral buffered formalin and processed using histological techniques. The paraffin sections were smeared with hematoxylin-eosin (HE) for histological examination using light microscopy, and the immunohistochemical slides were prepared.

The immunohistochemical reaction for quantifying parasitism was performed using the Novolink® kit (Novocastra® RE7260-K), according to the protocol described by Moreira *et al* ^15^ and Rossi *et al* ^16^, with slight modifications. The tissues embedded in paraffin were deparaffinized and rehydrated. For antigen retrieval, slides were placed in a solution of citric acid (10 mM, pH 6.0) at 95°-99°C for 30 minutes in a water bath. Endogenous peroxidase activity was blocked with a 3% hydrogen peroxide solution (six exchanges of 5 minutes each, in the dark). Then, non-specific ionic interactions were blocked by incubating the slides with 6g per 100mL of phosphate-buffered saline (PBS) of skim milk powder (Molico®, Nestlé, São Paulo, Brazil) for 1 hour at 37°C. Immunoblotting was performed by incubating the slides with an anti-Leishmania polyclonal antibodies produced in mice, diluted 1:1000 in 1% bovine albumin solution in PBS (0.01 M) containing 1% bovine serum albumin (BSA), in a humid chamber for 1 hour at 37°C. After washing, the sections were incubated with the Novolink kit post-primary blocking reagent (Novocastra® RE7260-K) for 1 hour at 37°C, and washed three times in PBS-Tween for 5 minutes. Then, the sections were incubated for 45 minutes at 37°C with the Novolink® polymer (Novocastra® RE7260-K), washed, and revealed using chromogenic substrate DAB + H2O2 (diaminobenzidine with hydrogen peroxide, DakoCytomation® K3468, Dako Denmark A/S). Sections were contrasted with Harris hematoxylin, dehydrated, and mounted with resin and glass coverslips. A positive control (patient skin with American cutaneous leishmaniasis) and a negative control (omission of primary antibodies) were used in the immunohistochemical assay. Parasitism in the tissue was analyzed by two independent pathologists through quantitative morphometric analysis, according to Laurenti *et al* ^3^. Twenty different fields of each section were evaluated on a light microscope using the 40X objective and the number of amastigotes per high power field (measuring 0.5mm in diameter, 0.196mm2) was determined using a Nikon Eclipse E200 model microscope. Tissue parasitism was considered negative when no parasite was visualized in 20 fields, low parasitism when one to ten amastigotes per field were visualized, moderate parasitism when 11-25 amastigotes per field were visualized, and high parasitism when more than 25 amastigotes per field were visualized. To avoid false-negatives, the slides of the negative cases were thoroughly reviewed by the pathologists.

Statistical analysis was performed using Stata/SE® 10.0 for Windows (College Station, Texas, USA).

This study was approved by the Ethics Committee in Research of the Federal University of Piauí (Approval No.1806554). All participants signed Free and Informed Consent Forms or the Free and Informed Assent Forms.

## RESULTS

Twenty-two patients were included in the study. From these, 18 (81.8%) patients were diagnosed using the parasitological method (microscopy or culture) and four (18.2%) patients were diagnosed using only serological reactivity. The ages of the patients ranged from 5 months to 78 years of age (median: 30 years). Four patients (18.2%) were less than 2 years old and 16 (72.7%) were older than 18 years. In the children (under 12 years old), the number of male and female patients was equal. However 87.5% of the adults were male. The majority of the patients came from medium-sized cities in Piauí and Maranhão states and had low education levels (Table 1).

**TABLE 1:** Demographic characteristics of the study population. Teresina 2016-2017.

Characteristics	Number of patients (%)
Age	
0 to 23 months	04 (18.2)
2 to 18 years	02 (9.1)
> 18 years	16 (72.7)
Gender	
Female	05 (22.7)
Male	17 (77.3)
Origin	
Piauí	14 (63.6)
Maranhão	07 (31.8)
Pará	01 (4.6)
Estimated population of home city	
< 20.000 inhabitants	07 (31.8)
20.000 to 50.000 inhabitants	07 (31.8)
> 50.000 inhabitants	08 (36.4)
Schooling	
Illiterate	05 (22.7)
Elementary school	11(50.0)
Incomplete high school	01 (4.6)
Not applicable*	05 (22.7)
Anti-HIV serology	
Positive	07 (31.8)
Negative	15 (68.2)

*Children under five years old are not literate.

Seven patients (31.8%) were co-infected with HIV. Their T-CD4+ lymphocyte count ranged from 9 to 677 cells/mm^3^ (mean=222, median=49.5 cells/mm^3^). All of these patients were known to be HIV-infected prior to the diagnosis of VL, but two of them had not yet started anti-retroviral therapy.

**FIGURE 1:**
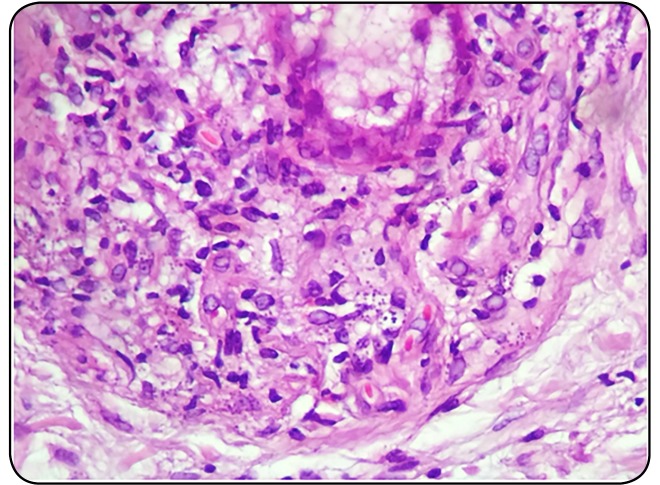
Hematoxylin-eosin revealing the presence of amastigotes in the skin of one of the patients under study (original magnification x400).

**FIGURE 2:**
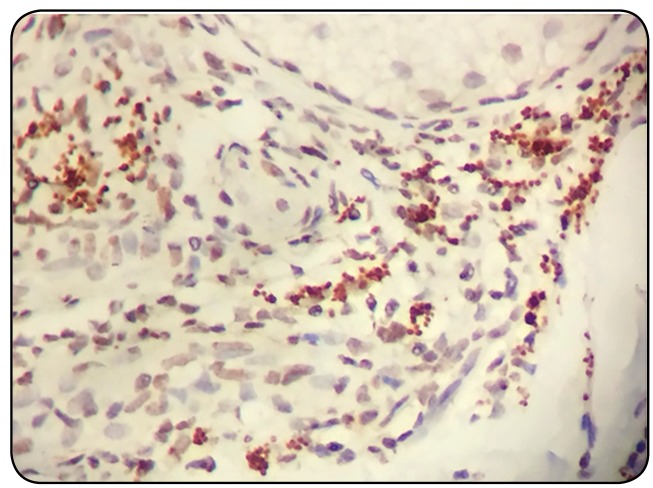
Immunohistochemistry evidencing positivity for anti-*Leishmania* antibody in the skin fragment of one of the patients under study (original magnification x400).

In 21/22 patients, no skin parasitism was observed through histopathology or immunohistochemistry. However, the biopsy specimen from one (4.5%) HIV/*Leishmania* co-infected patient was classified as high cutaneous parasitism (more than 50 amastigotes / field) (Figure 1 and Figure 2).

This patient was a 78 year-old man who was diagnosed with VL in January 2017. He reported pallor, apathy, increased abdominal volume, and coughing for 90 days, but had no referred fever, weight loss, edema, or bleeding disorder. He was diagnosed with immunodeficiency syndrome in September 2015 and had been on a highly active antiretroviral therapy since. His viral load was below the minimum detection limit since November 2016, but his T-CD4+ lymphocyte count performed before the diagnosis of VL, in August 2016, was 53 cells/mm^3^ (CD4+/CD8+=0.09), which was lower than his count at HIV diagnosis (140 cells/mm^3^, CD4+/CD8+=0.31).

He was in good overall health, had a preserved nutritional state, and had light pallor. His right retroauricular and cervical lymph nodes were approximately 0.5 cm in diameter, and splenomegaly and hepatomegaly were detected. There were no other signs or symptoms such as edema, bleeding, or jaundice.

Laboratory tests revealed his serum hemoglobin was 9.24 g/dL, leukocyte count was 4630/mm^3^, neutrophil count was 1343/mm^3^, platelet count was 147,000/mm^3^, serum albumin was 2.6 g/dL, globulin was 4.4 g/dL, serum creatinine was 0.9 mg/dL and urea was 31 mg/dL.

**FIGURE 3:**
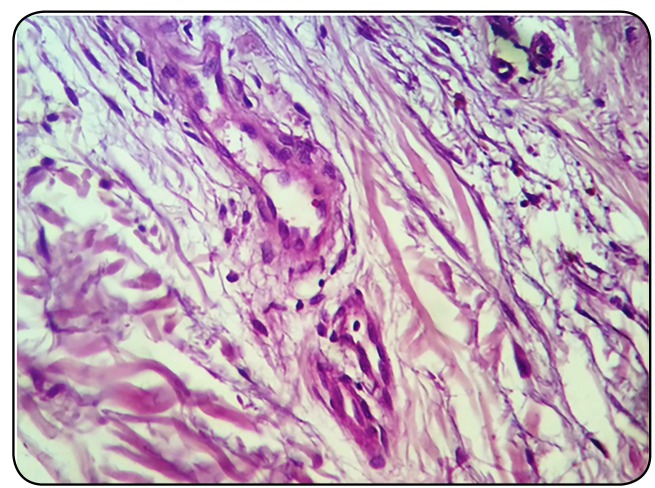
Hematoxylin-eosin staining revealing the absence of amastigotes in the skin fragment of one patient three days after the end of treatment (original magnification x400).

**FIGURE 4:**
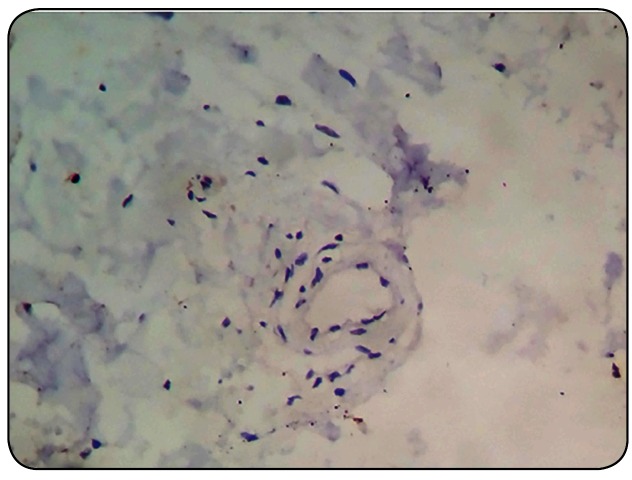
Immunohistochemistry evidencing negativity for anti-*Leishmania* antibody in skin fragment after three days of the end of treatment (original magnification x400).

The patient presented a positive rapid immunochromatographic test (rK39 antigen), but both direct amastigote screening and visualization of promastigotes in the bone marrow NNN medium were positive. He received liposomal amphotericin B (4mg/kg/day) for 14 days with a satisfactory response. Skin biopsies performed 72 hours after the end of treatment in the same forearm, adjacent to the pre-treatment biopsy, were negative for *Leishmania* staining (Figure 3 and Figure 4).

Secondary prophylaxis with liposomal amphotericin B every 14 days was prescribed for seven months. Laboratory post-treatment tests done in May 2017, three months after VL treatment, revealed a slight and non-significant improvement in T-CD4+ (70 cells/mm^3^) and viral load (127,414 copies/mL).

In August 2017, seven months after the termination of treatment, bone marrow amastigotes and *Leishmania* culture were negative and histopathological examination of the skin on the posterior forearm again did not show any signs of parasitism.

## DISCUSSION

The identification of a VL patient with high cutaneous parasitism raises the possibility that the anthroponotic transmission of *Leishmania* may occur in the Americas as it does in other areas of the world, albeit it may be rare. This individual had unsuccessfully been undergoing antiretroviral therapy for HIV for the two years prior to his diagnosis of VL. It is possible that this patient has been carrying a *Leishmania* infection for a long time. As people with HIV infection may have a clinical course of protracted VL, it is possible that they may act as reservoirs for the parasite for many months, perhaps even years, before VL is detected and the treatment of leishmaniasis is started.

Previous studies found proportions of VL patients with cutaneous parasitism in the skin varying from 0 to 38.9%. It is possible that this is partially due to differences in the method, but may also highlight the plausibility of man-phlebotomine-man transmission; thus, a new anthroponotic cycle may need to be considered in the epidemiology of *L. infantum* infection, especially in HIV co-infected patients. To our knowledge, this study is the first to have documented, by immunohistochemistry, the presence of cutaneous parasitism in individuals with active American VL and no visible skin lesions.

Molina *et al.* ^17^, using direct xenodiagnosis with *Phlebotomus perniciosus*, an important vector of VL, found that six patients in southern Europe co-infected with *Leishmania infantum* and HIV were able to infect sandflies. They determined that the infectivity of untreated patients was inversely proportional to their absolute T-CD_4_^+^ lymphocyte cell count.

The anthroponotic cycle of *Leishmania donovani* has been described in Asia and East Africa, as it is possibly associated with the high incidence of Post Kala-azar Dermal Leishmaniasis (PKDL). In Europe, the interhuman transmission of the parasite through shared contaminated syringes between individuals co-infected with HIV and *Leishmania infantum* has been described as one of the main forms of transmission in the region^18^. 

The ineffectiveness of culling *Leishmania*-infected dogs as a disease control program and the demonstration by xenodiagnostic studies that people with active VL, especially when co-infected with HIV^17^, can infect sandflies with *L. infantum*, strengthened the hypothesis that humans could be parasitic reservoirs^6^ ^,^ ^12^. In addition, the emergence of PKDL and cutaneous leishmaniasis caused by *Leishmania infantum* in endemic areas of VL^19^ ^,^ ^20^ reinforced the idea that cutaneous parasitemia can occur in American VL.

In Teresina, Piauí, asymptomatic individuals living in households where a recent case of active VL was diagnosed presented 71% more skin reactivity to *Leishmania*, representing one of the highest prevalence of reported asymptomatic infections^5^. In endemic areas, where people may be subjected to up to 30 bites of phlebotomines per hour, a symptomatic patient could infect almost 400 sandflies in 30 days, which is the mean time from VL symptom onset to fever and diagnosis^6^. This same study revealed that 25% of the individuals with active VL were able to infect at least one phlebotomine and 2.5% of the insects that fed on the VL patients were infected. In addition, they found that age (<4 years), diarrhea and peripheral blood neutrophil counts greater than 500/mL were independent predictors of infectivity. As the present study included few children, it is possible that cutaneous parasitism was underestimated in this population.

Other studies have already evaluated the infectivity of animals by sandflies. Deane & Deane^21^ observed that 75% of dogs, 28.5% of humans and a fox included in the study infected insects with VL. Interestingly, 24.8% of insects that fed on dogs became infected, while 14.8% of those who fed on humans and all insects that fed on the fox were infected. Therefore, while the chance of a sick dog infecting a phlebotomine is 25%, a fox can infect 100% and humans, only 15%. This study also noted that dogs without dermal parasitism were able to infect phlebotomines.

Recent experiments in *L. infantum*-infected dogs have demonstrated lasting parasitemia for over six months and the tendency of infections to remain on the skin around the site of the infectious sandfly bite^22^. Further, performing a biopsy in a covered area could result in a lower parasitic load and difficulty in visualization through immunohistochemical staining.

Previous cases of cutaneous parasitism by *Leishmania infantum* in humans with VL and apparently healthy skin have been previously described in Brazil, but there have not been any new publications on this topic in the last 20 years. In Pará, a study found rare parasites on the smear biopsy of healthy skin in one of the four individuals studied (25%)^11^.

In Ceará, Deane & Deane^21^ examined the dermal lymph in cutaneous lesions of 31 patients with kala-azar and found Leishmaniasis in a single patient, on the edge of a traumatic malleolar ulcer. In the same study, 76.3% of the dogs and 75% of the foxes had cutaneous parasitism in the smears.

In 1962, in a series of 43 patients with active VL and macroscopically normal skin, Deane & Deane found seven individuals with cutaneous parasitism (16.3%), one of which was very prominent. They showed that all patients whose skin had been parasitized during the visceral involvement were cured when re-examined after treatment^12^. However, the site of the biopsy and the exam that was used to detect the parasites is unclear. In the same study, the authors compared these results to canine cutaneous parasitism, which was abundant and frequent at the time (77.6% of dogs with VL had dermal parasitism).

Also in this state (CE), the researchers did not find any amastigote in tissue samples from biopsies of the subscapular paraspinal region of healthy skin from 27 individuals investigated, but identified promastigote forms in skin culture by means of monoclonal antibodies and enzymatic electrophoresis in seven (38.9%) of 18 patients studied^13^. However, the study did not identify whether the parasites were in the bloodstream or skin.

Prata & Piva^11^ biopsied apparently normal skin from the side of the arm or forearm of seven patients with VL in Bahia and observed the presence of *Leishmania* in the skin of one of them (14.3%). In Minas Gerais, investigators obtained a negative result for material obtained by scarification of the skin of all 23 studied patients^23^.

*Lu. longipalpis* is more attracted to humans than canine hosts^21^ ^,^ ^24^, and the human population is larger than the canine population in urban agglomerates; thus, it is expected that the number of infected humans is higher than the number of infected dogs. If so, although less likely to transmit the parasite to the vector, the importance of humans as reservoirs of *L. infantum* should not be neglected. Although asymptomatic carriers of *L. infantum* were less infectious for sandflies than individuals with active VL, their vast numbers in an endemic city would constitute a surprisingly large reservoir of parasites^6^.

The population enrolled in this study differs from the general population of people with VL due to the predominance of adults and HIV-infected men. The small sample of patients that could be included in the study restricts the power of generalization of the data. It is also possible that the proportion of people with VL presenting cutaneous parasitism due to *L. infantum* is greater than was found in this study.

In conclusion, amastigotes were viewed in histopathological and immunohistochemical examination on the skin of a patient with VL who was co-infected with HIV. Further studies are needed to prove whether humans infected with *Lutzomya longipalpis* in Brazil can transmit infections to other individuals systematically and effectively. 
